# Expert opinion on the use of transvaginal sonography for presurgical staging and classification of endometriosis

**DOI:** 10.1007/s00404-022-06766-z

**Published:** 2022-11-11

**Authors:** J. Keckstein, M. Hoopmann, E. Merz, D. Grab, J. Weichert, S. Helmy-Bader, M. Wölfler, M. Bajka, S. Mechsner, S. Schäfer, H. Krentel, G. Hudelist

**Affiliations:** 1 Endometriosis Clinic Dres, Jörg und Sigrid Keckstein, Richard Wagner Strasse18, Villach, Austria; 2 Department of Obstetrics and Gynaecology, Medical University Ulm, Ulm, Germany; 3 Department of Obstetrics and Gynaecology, Medical University Tübingen, Tübingen, Germany; 4 Centre for Ultrasound and Prenatal Medicine, Frankfurt, Germany; 5 Department of Obstetrics and Gynaecology, University Hospital of Schleswig-Holstein, Lübeck, Germany; 6 Department of Obstetrics and Gynaecology, Medical University Vienna, Vienna, Austria; 7 Department of Obstetrics and Gynaecology, Centre for Endometriosis, Medical University Graz, Graz, Austria; 8 OB/GYN Volketswil, Volketswil, Switzerland; 9 Department of Gynaecology, Endometriosis Centre Charité, Charite Berlin University Hospital, Berlin, Germany; 10 Department of Gynaecology and Obstetrics, University Hospital Muenster, Münster, Germany; 11 Department of Obstetrics and Gynaecology, Bethesda Hospital Duisburg, Duisburg, Germany; 12 Department of Gynaecology, Centre for Endometriosis, Hospital St. John of God, Vienna, Austria; 13 Rudolfinerhaus Private Clinic and Campus, Vienna, Austria; 14 SEF, Scientific Endometriosis Foundation (Stiftung Endometrioseforschung), Westerstede, Germany; 15 AGEM, Arbeitsgemeinschaft Endometriose of the DGGG, Berlin, Germany; 16 ÖGUM, Österreichische Gesellschaft für Ultraschall in der Medizin, Vienna, Austria; 17 SGUM, Schweizer Gesellschaft für Ultraschall in der Medizin, Aarau, Switzerland; 18 EEL, European Endometriosis League, Unterhaching, Germany

**Keywords:** Endometriosis, Ultrasound, Diagnostics, #Enzian classification, Recommendation

## Abstract

Gynecological ultrasonography plays a central role in the management of endometriosis. The rapid technical development as well as the currently increasing evidence for non-invasive diagnostic methods require an updated compilation of recommendations for the use of ultrasound in the management of endometriosis. The present work aims to highlight the accuracy of sonography for diagnosing and classifying endometriosis and will formulate the present list of key messages and recommendations. This paper aims to demonstrate the accuracy of TVS in the diagnosis and classification of endometriosis and to discuss the clinical applications and consequences of TVS findings for indication, surgical planning and assessment of associated risk factors. (1) Sophisticated ultrasound is the primary imaging modality recommended for suspected endometriosis. The examination procedure should be performed according to the IDEA Consensus. (2) Surgical intervention to confirm the diagnosis alone is not recommended. A preoperative imaging procedure with TVS and/or MRI is strongly recommended. (3) Ultrasound examination does not allow the definitive exclusion of endometriosis. (4) The examination is primarily transvaginal and should always be combined with a speculum and a bimanual examination. (5) Additional transabdominal ultrasonography may enhance the accuracy of the examination in case of extra pelvic disease, extensive findings or limited transvaginal access. (6) Sonographic assessment of both kidneys is mandatory when deep endometriosis (DE) and endometrioma are suspected. (7) Endometriomas are well defined by sonographic criteria. When evaluating the ovaries, the use of IOTA criteria is recommended. (8) The description of sonographic findings of deep endometriosis should be systematically recorded and performed using IDEA terminology. (9) Adenomyosis uteri has sonographically well-defined criteria (MUSA) that allow for detection with high sensitivity and specificity. MRI is not superior to differentiated skilled ultrasonography. (10) Classification of the extent of findings should be done according to the #Enzian classification. The current data situation proves the best possible prediction of the intraoperative situs of endometriosis (exclusive peritoneum) for the non-invasive application of the #Enzian classification. (11) Transvaginal sonographic examination by an experienced examiner is not inferior to MRI diagnostics regarding sensitivity and specificity in the prediction of the extent of deep endometriosis. (12) The major advantage of non-invasive imaging and classification of endometriosis is the differentiated planning or possible avoidance of surgical interventions. The recommendations represent the opinion of experts in the field of non-invasive and invasive diagnostics as well as therapy of endometriosis. They were developed with the participation of the following national and international societies: DEGUM, ÖGUM, SGUM, SEF, AGEM/DGGG, and EEL.

## What does this study add to the clinical work

**Table:** 

The TVS is an efficient, accurate, and cost-effective tool for the non-invasive diagnosis of endometriosis. #Enzian classification for the description of TVS correlates very well with surgical findings, and will provide clinicians with a standardized language for the comprehensive description of endometriosis.	

## Introduction

### Sophisticated ultrasound is the primary imaging modality recommended for suspected endometriosis: the examination procedure should be performed according to the IDEA consensus

First described in 1860 [1] and primarily diagnosed via bimanual palpation performed before surgery and histological confirmation, endometriosis can now be described with high accuracy via several non-invasive imaging methods. Today, ovarian endometriomas and deep endometriosis (DE) can be detected by ultrasound or magnetic resonance imaging (MRI) [2–5]. In addition, adhesions can also be visualized indirectly using organ mobility and sliding signs on transvaginal sonography (TVS) [6, 7]. Accurate sonographic evaluation of the different forms of endometriosis has become one of the most important elements in the management of affected women, which is now included in the recommendations of the national and international societies [8–12]. However, the former lack of standardized definitions in the sonographic classification and divergent methods of classifying the affected anatomical location and extent of the disease led to evident and inconclusive variations in the reported diagnostic accuracy of TVS in the diagnosis of endometriosis. This problem was addressed by the International Deep Endometriosis Analysis (IDEA) group in 2016. They proposed a systematic approach for sonographic workflow and specified terms, definitions, and measurements to document the dimension and location of the lesions [3].

This IDEA Consensus is the most widely used and accepted standard for the sonographic examination procedure in patients with endometriosis [13].

### Surgical intervention to confirm the diagnosis alone is not recommended: a preoperative imaging procedure with TVS and/or MRI is required

Although surgery is still considered the diagnostic gold standard, especially in patients with the peritoneal disease, this dogmatic approach brings three major problems that need to be discussed. First and foremost, surgical and subsequent histologic diagnosis is again based on the surgeon’s visualization of endometriosis. Extensive adhesions and deep endometriosis (DE), some of which may be extraperitoneal, may primarily obscure the extent of the disease. Dissection of the occluded spaces requires experience and advanced surgical skills of the surgeon to meet the requirements of a “gold standard test.” As a result, patients with severe adhesions or a so-called “frozen pelvis” may underestimate the true extent of endometriosis. Especially in patients with minor symptoms, the indication for surgery and in particular the extent of the procedure must be weighed against the potential risks [14]. Second, visualization of disease—even in the case of minor peritoneal endometriosis—is by nature subjective. Hence, there is some evidence that surgical subjectivity may lead to relevant discrepancies in final diagnosis and may even poorly correlate with histological proof of the disease, especially under non-tertiary referral, and routine conditions [15].

Third, uterine adenomyosis cannot always be confirmed visually or even histologically in patients with fertility problems, which may lead to a diagnostic dilemma regarding the laparoscopic “gold standard test.” As a consequence, the eminent European Society for Human Reproduction (ESHRE) states in the updated and probably most extensive and most cited endometriosis guideline regarding laparoscopic identification of endometriosis as a gold standard test that *“…advances in the quality and availability of imaging modalities for at least some forms of endometriosis on the one hand and the operative risk, limited access to highly qualified surgeons and financial implications on the other, calls for the urgent need for a refinement of this dogma *[9]*, and delete the diagnostic laparoscopy as recommended gold standard in the diagnosis of endometriosis, when imaging finding shows changes suspected of endometriosis”.*

Diagnostic imaging methods include multiple modalities such as MRI, computed tomography (CT), X-ray and sonography. Regarding the non-invasive diagnosis of endometriosis, only MRI and sonography in form of TVS have been proven reliable and accurate tools for diagnosing the disease [5, 16].

### Ultrasonography does not allow the definite exclusion of endometriosis

A Cochrane Review concerning the imaging modalities for endometriosis concludes that TVS and MRI help surgeons to better plan an operative procedure [17]. However, the authors also state that none of the imaging techniques was accurate enough to ensure complete detection of total pelvic endometriosis. Superficial peritoneal endometriosis may be the only entity which cannot be reliably diagnosed by any imaging method [18]. A recently published prospective, multicentre study including 745 patients undergoing TVS and surgery found excellent sensitivities for DE and ovarian endometriosis [19]. But none of the analyzed anatomical sites reached a 100% detection rate. The lowest rates were described for extrapelvic nodules such as DE in the intestine (above the rectosigmoid colon), diaphragm, lung, or nerves.

The different accuracy of sonographic diagnostics is mainly influenced by the size and localization of the findings and the accessibility with the ultrasound probe, but also by the skill and experience of the examiner. Learning the technique requires a certain number of examinations, as Tammaa [20] demonstrated in Douglas obliteration and DE on the rectum.

The present work should contribute as a basis for the establishment of standardized sonographic diagnostics of endometriosis, which in the future should affect the standards of training and required quality of diagnostics.

### The examination is primarily transvaginal and should always be combined with a speculum and a bimanual examination

TVS has proven to be a cost-effective, easy-at-hand tool showing real-time assessment of the uterus, the pouch of Douglas, and ovaries. In addition, the visualization and assessment of the ureters, urinary bladder, and rectum facilitate the diagnosis of anatomical changes due to endometriosis. Compared to bimanual examination, several papers have shown the diagnostic superiority of TVS. However, especially in patients with vaginal lesions, the combination of imaging techniques and clinical examination, including speculum examination and bimanual palpation, leads to a clearer view of the structures involved [21–23]. In addition, the dynamic examination includes not only imaging of endometriosis but also assessment of motility of the pelvic organs (sliding signs), tissue elasticity, and tenderness of affected organs (compartments) [6, 24].

### Additional transabdominal ultrasonography may enhance the accuracy of sonography in case of extrapelvic disease, extensive findings or limited transvaginal access

**Fig. 1:**
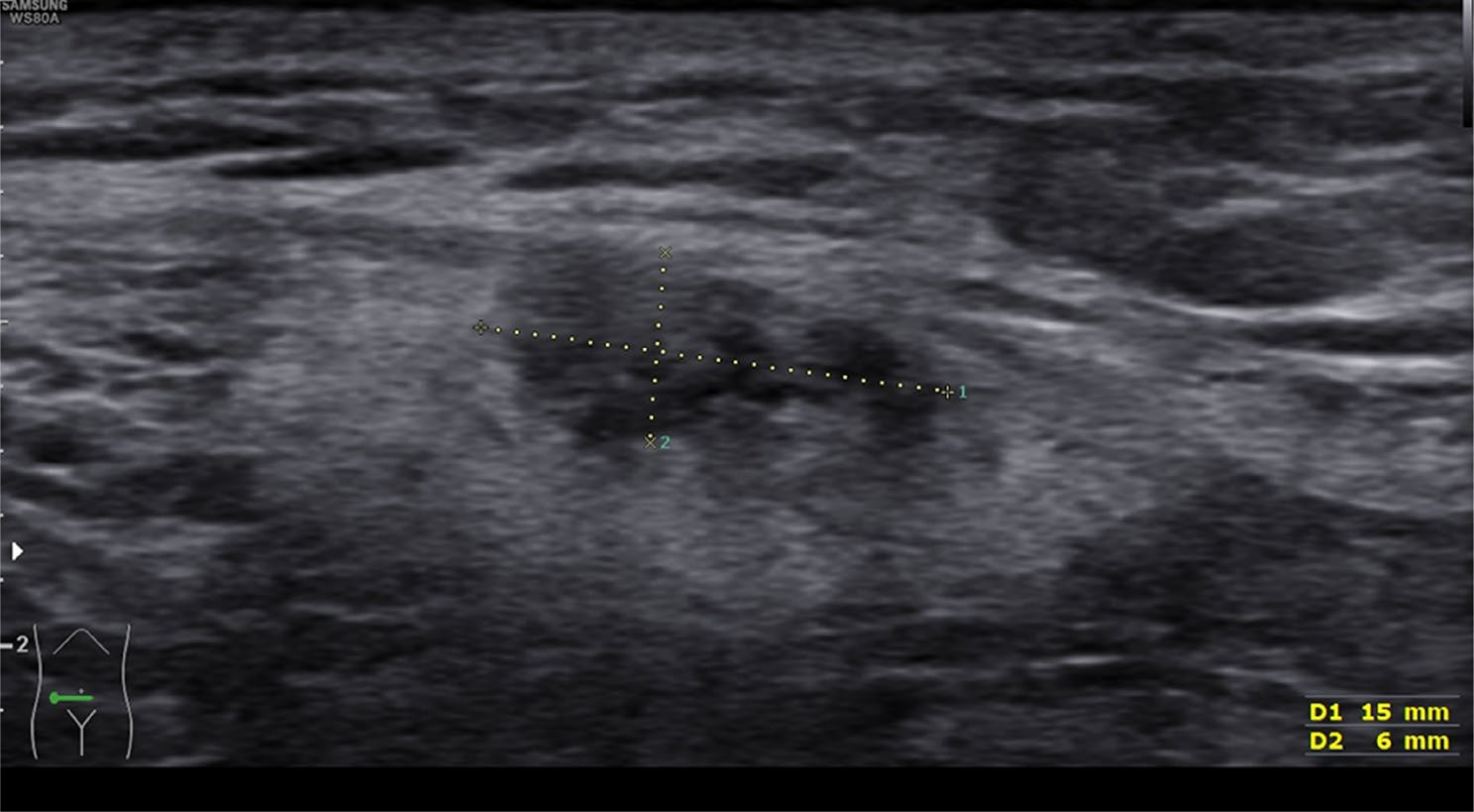
Transabdominal ultrasound to identify a deep endometriosis nodule in the abdominal wall (#Enzian(u) FOabd. wall)

Higher resolution and anatomical proximity are key advantages of TVS for a pelvic examination, but in cases with the severe extent of DE, lesions can exceed beyond the pelvic region (e.g., in higher sections of the intestine, abdominal wall, or diaphragm, Fig. 1). In these cases, transabdominal sonography can help to complete the anatomical evaluation. Furthermore, in some patients, the favored vaginal ultrasound access is not feasible (for example, due to vaginal stenosis or vaginismus), so the transabdominal route can be used as an alternative. The performance of abdominal sonography is primarily based on symptoms. However, sonography of the kidneys is also essential in asymptomatic deep endometriosis.

#### Assessment of the kidneys by transabdominal sonography is mandatory when deep endometriosis and endometriomas are suspected

Hydronephrosis is a common and relevant complication of DE, especially in cases with ureteral endometriosis. Subjective urinary tract symptoms may be present, but a silent loss of kidney function occurs in a significant part of the patients. Transabdominal ultrasound is an easy and reliable method for detecting and evaluating hydronephrosis [25]. In case of endometrioma, the probability of concomitant DE of the pelvic wall is high and needs also more extensive attention [6].

### Endometriomas are well defined by sonographic criteria: when evaluating the ovaries, the use of IOTA criteria is recommended

**Fig. 2:**
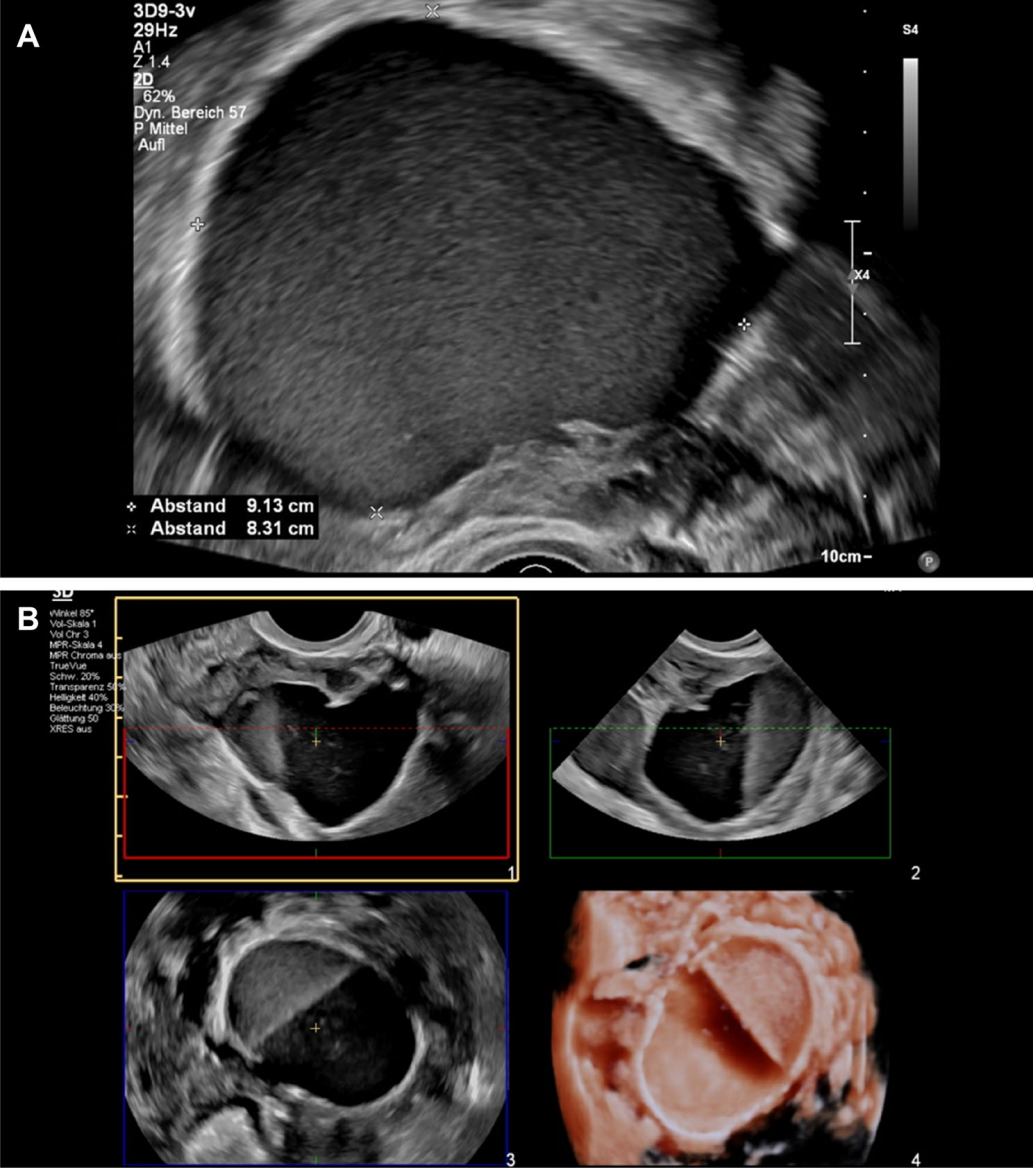
**a** Typical unilocular endometrioma (diameter > 7 cm = #Enzian(u)O3). Echogenicity: ground glass-like echogenicity. **b** Sonographic image of an atypical multilocular ovarian endometrioma with different echogenicity of the locules

**Fig. 3:**
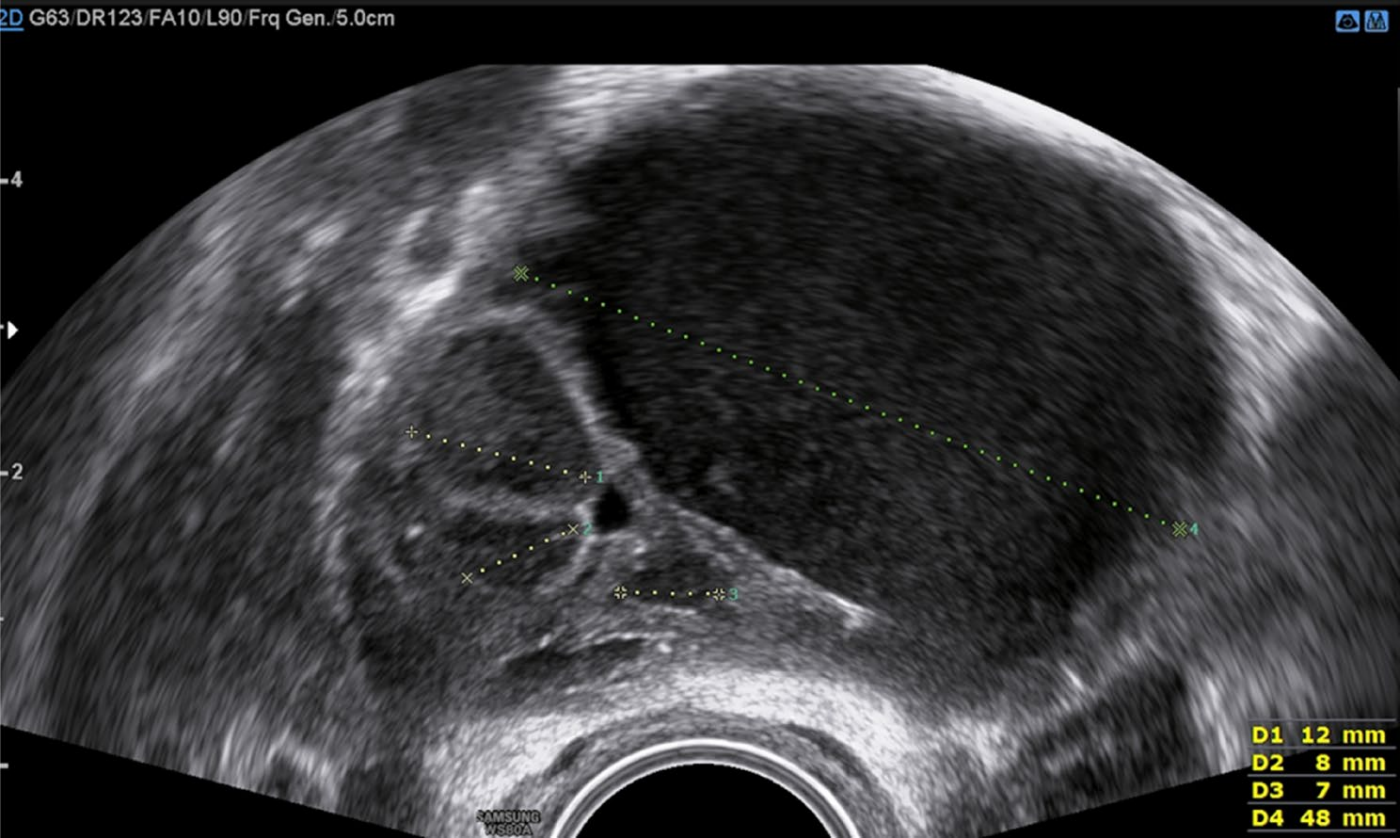
Multilocular endometrioma (sum of all diameters 6.5 cm = #Enzian(u)O2)

Regarding the diagnosis of ovarian endometriomas, a Cochrane review on non-invasive tests for diagnosis of endometriosis by Nisenblat et al. [26] summarizes 8 studies including 765 patients with endometriomas demonstrated an overall sensitivity and specificity of 93% and 96%, respectively. Endometriomas are among the most common preoperative findings of adnexa with a pathognomonic sonomorphologic appearance. The international ovarian tumor analysis (IOTA) group has, therefore, summarized the typical picture of endometriomas as benign simple descriptor: unilocular tumor with ground-glass echogenicity in a premenopausal woman (Fig. 2a, b) [27]. This is the most common but not the only presentation of endometriomas. They can also be multilocular (Fig. 3), but then they do not have more than four cysts. Papillary projections are found in 10%, but most often without internal blood flow. Occasionally, peripheral punctate echogenic foci and sludge are seen with endometriomas. CA125 may be moderately elevated (median 44 U/mL) [28]. Mascilini’s study [29] showed that it is possible to distinguish decidualized endometriomas with papillary projections from borderline tumors with papillary features by assessing the contour of the papillary projection and the echogenicity of the cyst fluid. This differentiated description can significantly reduce the number of unnecessary surgeries for adnexa during pregnancy.

### The description of sonographic findings of deep endometriosis should be systematically recorded and performed using IDEA terminology

DE is a particular form of endometriosis that penetrates more than 5 mm under the peritoneal layer thereby causing typical sonoanatomical changes in affected organs such as the urinary bladder, vagina, parametrial tissues, and intestines [30]. The IDEA criterion additionally differentiates the depth of infiltration into the affected organs [3]. There is good evidence that there is a direct correlation between the extent of DE and the severity of symptoms [31].

TVS has been recommended as the first-line diagnostic tool to assess patients with suspected DE [32, 33]. Although the utility of TVS for diagnosing DE is proven, it should be discussed that the method is strictly operator-dependent. Consequently, TVS performed by an untrained and/or non-gynecologic operator has limited diagnostic potential. Thus, high-quality TVS is limited to experienced sonographers and/or certified tertiary referral centers [20, 34].

**Fig. 4:**
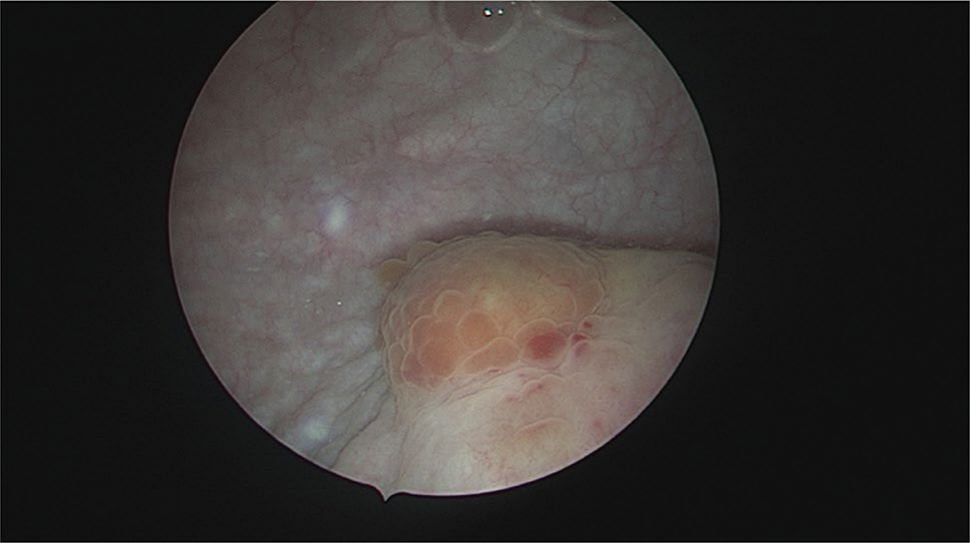
Cystoscopic view of a deep endometriosis nodule in the posterior bladder wall (#Enzian(s)FB)

**Fig. 5:**
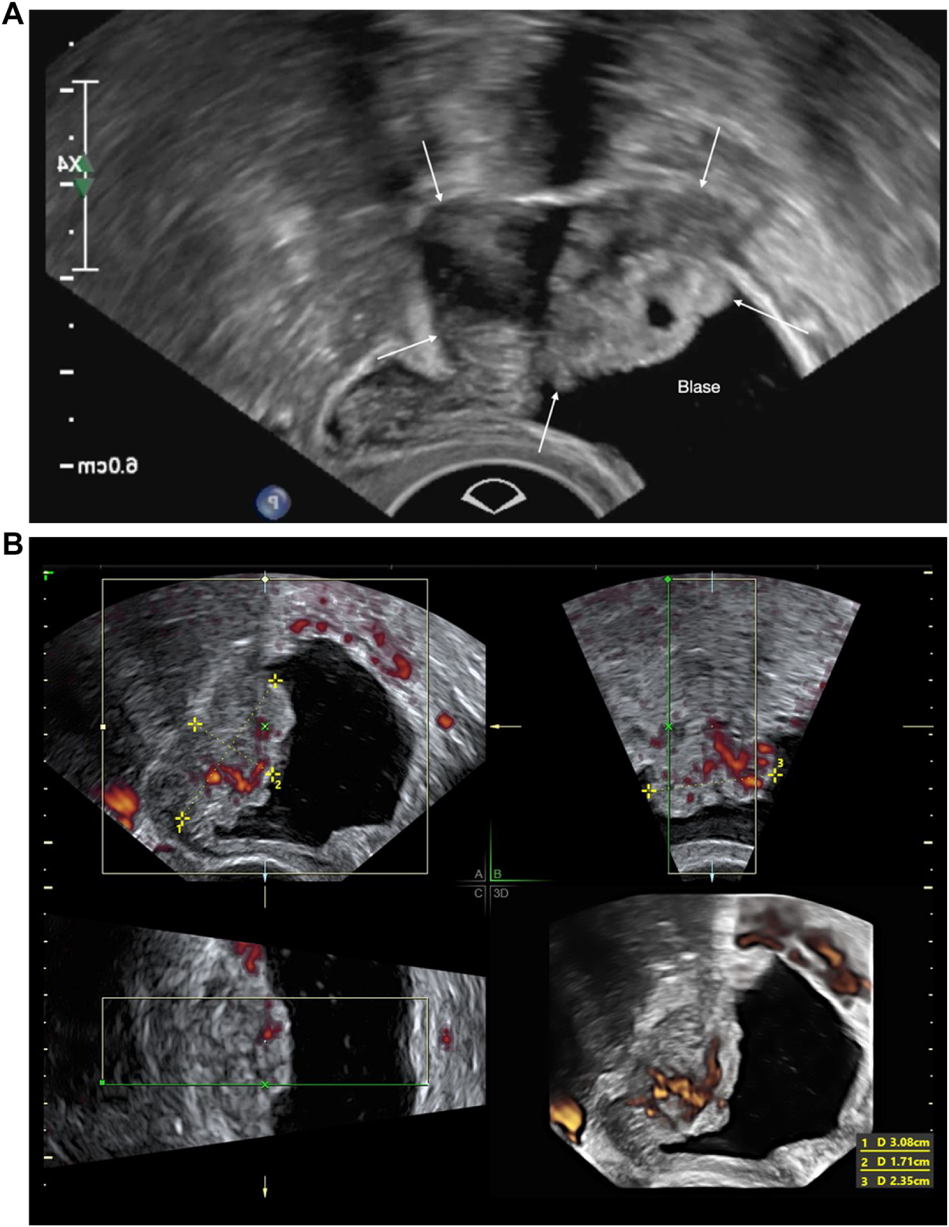
**a** Sonographic view of DE of the bladder (#Enzian(u)FB), presenting a full thickness defect at the bladder dome by a large inhomogeneous deep endometriosis nodule. **b** 3D demonstration of a severe bladder endometriosis (30.8 × 17.1 × 23.5 mm) originating from the posterior bladder wall (#Enzian(u)FB). A–C Multiplanar glass body demonstration (grey scale + color Doppler): A = sagittal view, B = transverse view, C = coronal view. 3D = surface demonstration of the cut plane with monochromic demonstration of the vascularization

**Fig. 6:**
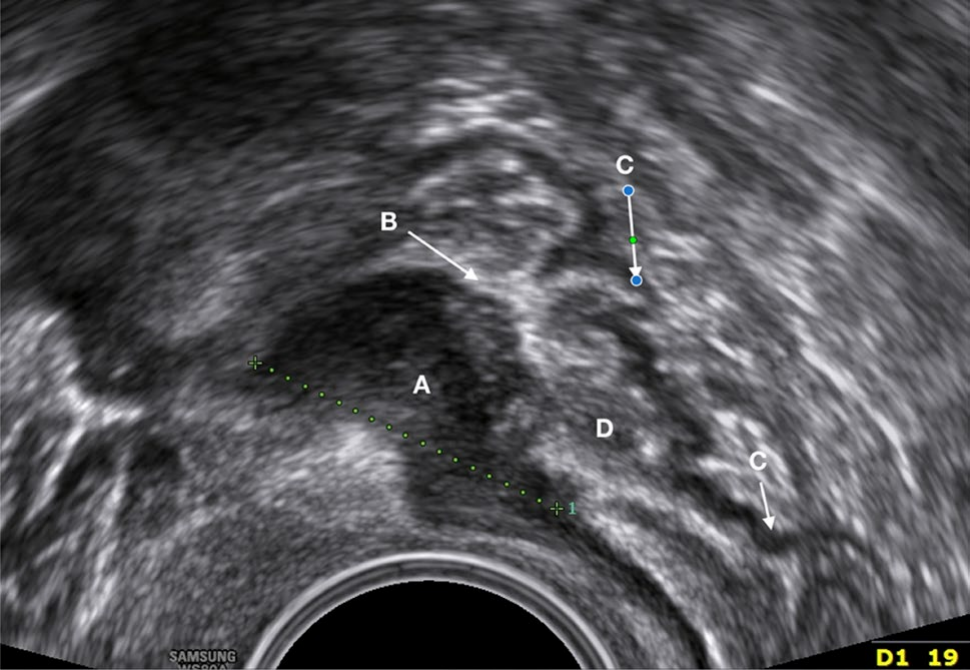
Transvaginal image of a deep rectal endometriosis nodule (length 1.9 cm = #Enzian(u)C2). A = thickened intestinal muscle layer with deep nodule (hypodense), B = mucosal layer (hyperdense), C = muscle layer of the posterior rectal wall (hypodense), and D = the lumen of the intestine

**Fig. 7:**
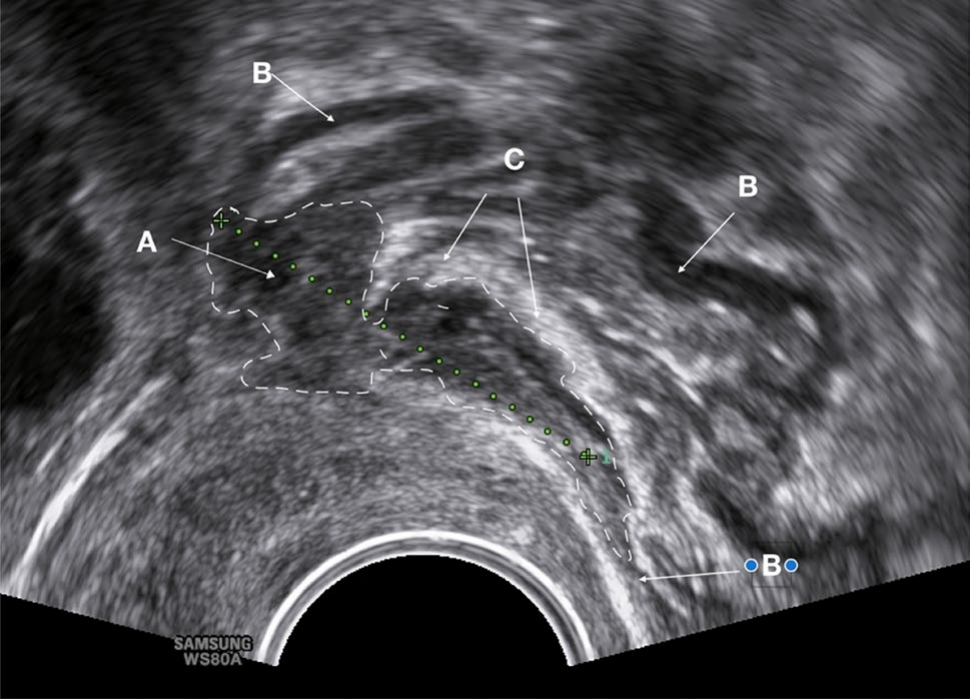
Sonographic image of an irregular deep endometriosis nodule in the rectal anterior wall (length 1.8 cm = #Enzian(u)C2). The hypodense area (A) represents the marked widening of the muscle layer due to a deep endometriosis nodule, accompanied by fibrosis or myohyperplasia of the layer. The normal pattern of the muscle layer is visible in the caudal direction and the parts of the posterior wall (B). The lesion lies directly underneath the mucosa (full thickness defect) (C)

**Fig. 8:**
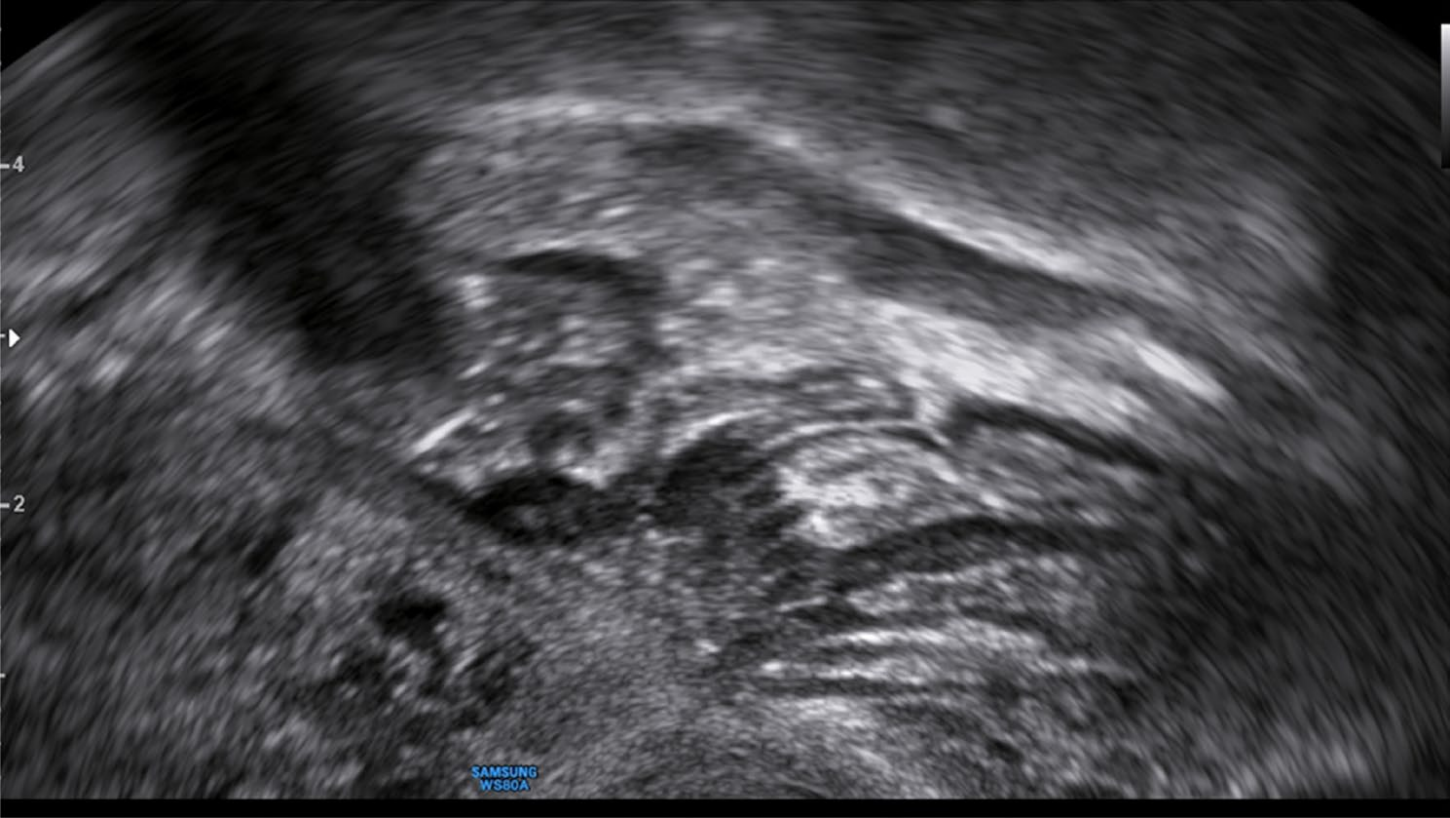
Image of a deep nodule presenting a full thickness defect in the anterior rectum wall with prominent spikes towards the bowel lumen with extrinsic reaction (hypodense area; zig-zagged shaped)

**Fig. 9:**
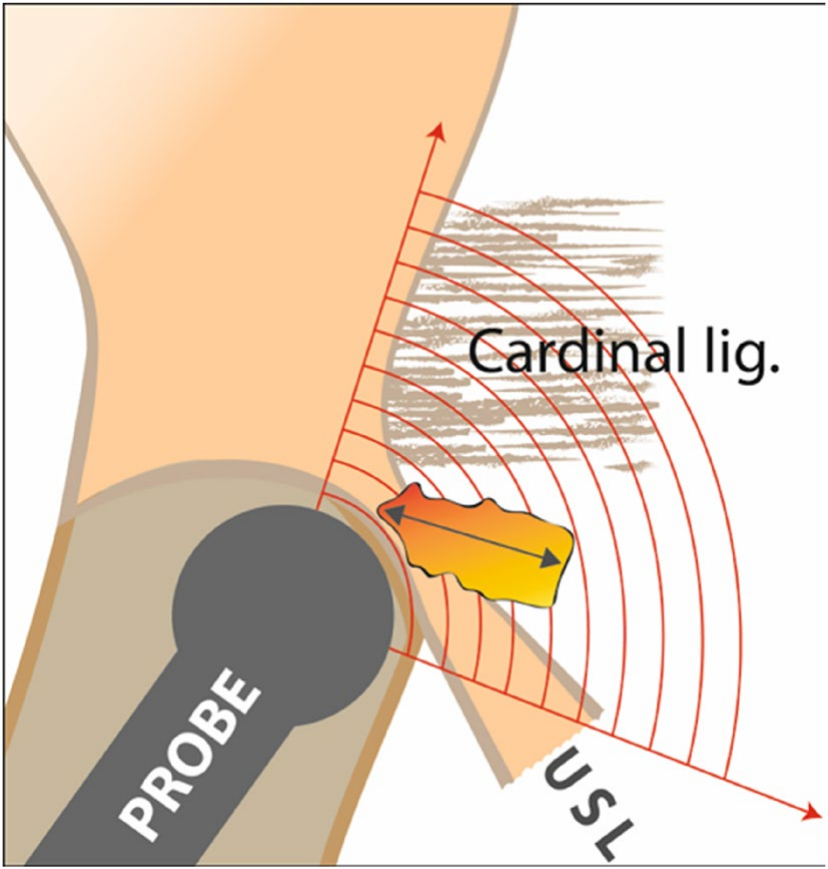
Schematic drawing of the ultrasound probe position for exact evaluation of the uterosacral ligaments and the parametrium (= #Enzian B compartment). The probe is moved slightly laterally in the uterine fornix and then tilted between 20 and 90 degrees

**Fig. 10:**
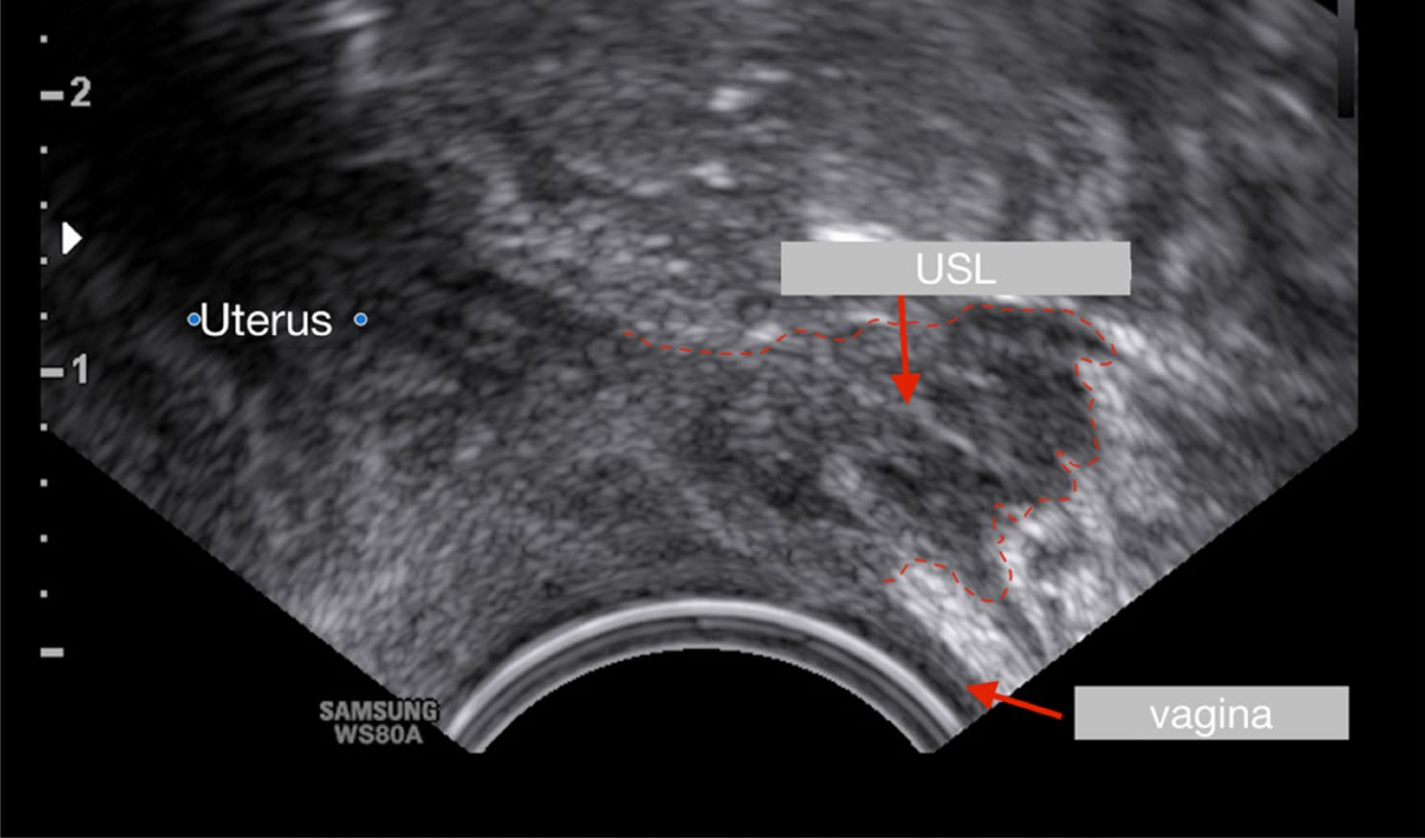
Sonographic image (hypodense areal) of the right uterosacral ligament (USL) (length 1, 2 cm = #Enzian(u)B0/2). The ligament is infiltrated by endometriosis and significantly thickened. In the left part of the image, parts of the cervix uteri are also visible. The vaginal wall is sonographically inconspicuous and has a normal thickness

To create uniform terms and definitions for DE and TVS in combination with a structured protocol on how to assess and document DE with TVS, the International Deep Endometriosis Analysis (IDEA) group published a consensus statement in 2016 [3]. As the first one of its kind, it provides clinicians with concise definitions of DE visualized on TVS and allows for a structured step-by-step assessment of pelvic organs of the so-called anterior with urinary bladder (Figs. 4, 5a, b) and ureters and posterior compartment (intestines, uterosacral ligaments, rectovaginal septum and vagina, Figs. 6, 7, 8, 9, 10).

The high diagnostic accuracy of TVS for diagnosing DE is well documented. In their Cochrane review, Nisenblat et al. [26] report a mean sensitivity of 79% (95% CI 69–89%) and specificity of 94% (CI 88–100%) for TVS-based diagnosis of DE, thereby fulfilling the criteria of a triage test to rule in endometriosis. So far, additional four systematic reviews and meta-analyses have examined the validity of TVS for diagnosing DE over the past decade [2, 5, 35, 36]. Following previous works, recently published pooled sensitivities and specificities for colorectal DE are 89% and 97% and 55% and 99% for DE affecting the urinary bladder with relevant heterogeneity of the reviewed studies on this anatomical location. Notably lower values were observed for uterosacral DE with a sensitivity of 64% (95% CI 50–79%) and specificity of 97% (93–100%) [17], which is in line with the recently published work by Gerges et al. [5].

### Adenomyosis uteri has sonographically well-defined criteria (MUSA) that allow for detection with high sensitivity and specificity: MRI is not superior to a differentiated experienced ultrasound examination

Adenomyosis uteri is defined as the presence of ectopic, non-neoplastic endometrial glands and stroma within the myometrium. As a rule, the ectopic endometrium is surrounded by hypertrophic and hyperplastic myometrium. In severe cases, the entire structure of the myometrium, i.e., the architecture of the uterine wall, is completely destroyed. Especially young patients with adenomyosis uteri frequently suffer from pain and dysmenorrhea. Furthermore, adenomyosis uteri affects the reproductive outcome and leads to pregnancy and obstetrical complications [37]. Therefore, diagnosing adenomyosis as early as possible is crucial with non-invasive imaging techniques. For decades, adenomyosis could only be reliably diagnosed by performing a targeted biopsy or hysterectomy and histopathological analysis of the tissue. It is only since the 1980s, with the advent of high-resolution ultrasound and the development of magnetic resonance imaging (MRI), that the diagnosis of adenomyosis can be made accurately and with sufficient sensitivity without the need for surgery or removal of the uterus. Knowledge of the imaging criteria is critical in this regard. The manifestations are heterogeneous, but typical criteria of adenomyosis uteri are [38]:

- globally enlarged uterus
- asymmetry between the anterior and posterior wall of the uterus
- irregular and/or ill-defined lesions without rim
- fan-shaped shadowing
- non-uniform, mixed echogenicity with cysts, hyperechogenic islands and/or sub-endometrial lines and buds
- in Doppler sonography depiction of a translesional flow
- the junctional zone is often thickened, irregular or ill-defined
- depictable interruption of the junctional zone

**Fig. 11:**
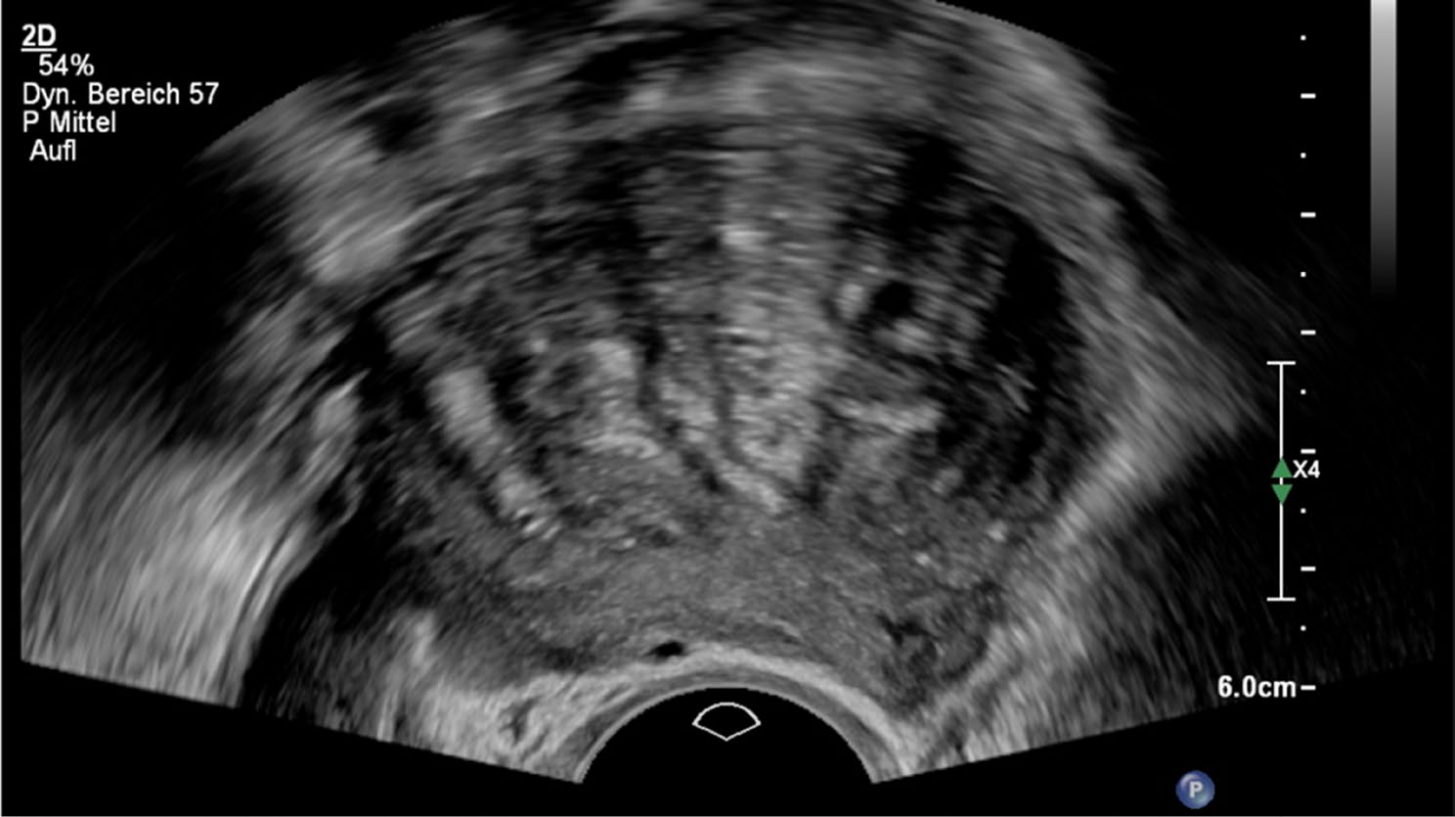
Adenomyosis (#Enzian(u)FA): asymmetry between the anterior and posterior wall of the uterus; fan-shaped shadowing non-uniform; mixed echogenicity with cysts, hyperechogenic islands

**Fig. 12:**
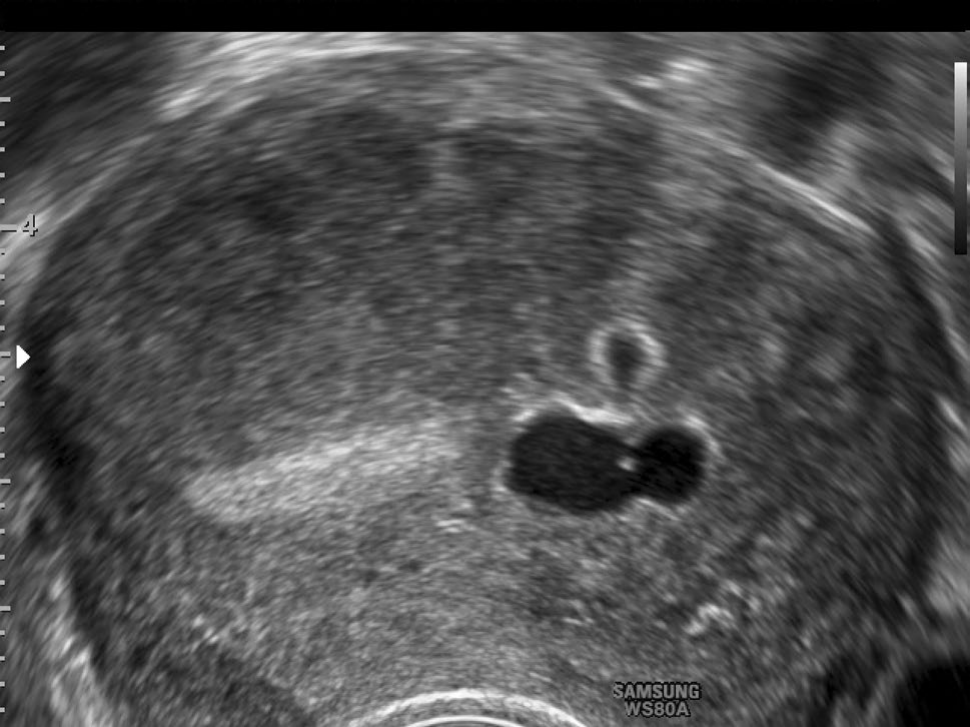
Cystic adenomyosis (#Enzian(u)FA). Typical signs: asymmetry; mixed echogenicity; sub-endometrial cystic lesion

Usually, not all the above criteria are met at the same time (Figs. 11, 12). The terms and definitions have been standardized and described in a consensus paper of the Morphological Uterine Sonographic Assessment (MUSA) group [38]. These diagnostic criteria are also anchored in the current quality requirements for DEGUM level 2 for gynecological sonography [11]. The diagnostic accuracy of TVS is high. In a recent meta-analysis, the sensitivity and specificity were 78% (AUC 0.73) [39]. The combination of 2D and 3D ultrasound tended to improve diagnostic accuracy. The so-called question mark sign describes the position of the uterus fixed in retroflection [3]. Adhesions and DE lesions primarily cause this. However, the sonographic picture of the question mark sign also correlates with adenomyosis uteri. In a further meta-analysis, the addition of the sonographic question mark sign leads to an ameliorated overall sensitivity and specificity of transvaginal ultrasound, which was 83% and 88%, respectively [40]. No diagnostic superiority of MRI could be found, so transvaginal sonography is recommended as a first-line method due to its better availability and lower costs [39, 41].

### Classification of the extent of findings should be done according to the #Enzian classification: the current data prove the best possible prediction of the intraoperative situs of endometriosis (exclusive peritoneum) for the non-invasive application of the #Enzian classification

The accurate documentation can be done individually (description) or in a standardized form, e.g., by a uniform classification. This is of great advantage both for the rapid assessment of the findings and for interdisciplinary communication.

The ideal system for classifying endometriosis should be applicable for imaging and surgical interventions. Although several scores and systems have been proposed over the past 50 years [42], the main surgical classification systems which are currently used in everyday clinical practice worldwide are the rASRM score [43], the Enzian classification [44] and the so-called EFI (endometriosis fertility index) [45] which is rather a prediction model for fertility purposes following surgery for endometriosis. Finally, the American Association of Gynaecological Laparoscopists (AAGL) recently proposed the so-called AAGL score for surgical staging and description of endometriosis [46]. To date, several studies have tried to evaluate the use of TVS in combination with the rASRM and Enzian classification. High-quality studies on the applicability of TVS with other classification systems are lacking so far.

The rASRM classification, which has been in use over decades, primarily focuses on the effects of endometriosis on fertility in association with peritoneal and ovarian disease and secondary adhesions. This excludes the detailed description of DE which is considered the main disadvantage of this score [44, 47]. Nevertheless, there have been attempts to use TVS in combination with the rASRM score. In a retrospective study including 204 women, Leonardi et al. [48] found the accuracy of TVS for the prediction of the surgical rASRM stage to be 53.4% for stage 1, and 93.8%, 89.7% and 93.1% for stages 2, 3 and 4, respectively. Sensitivities, specificities, positive predictive values (PPVs) and negative predictive values (NPVs) of TVS were 18.2%, 94.7%, 80% and 49.7% for rASRM stage 1, 22.7%, 96.7%, 45.5% and 91.2% for stage 2, 62.5%, 92.0%, 40.0% and 96.7% for stage 3 and 71.9%, 97.1%, 82.1% and 94.9% for stage 4, suggesting higher accuracy for TVS in higher disease stages. In a prospective study including 201 women, Holland et al. [7] also found good agreement between TVS findings and the surgical rASRM stage (absent, minimal, mild, moderate, and severe endometriosis; quadratic weighted kappa = 0.786). However, they also observed low sensitivity for TVS diagnosing minimal and mild endometriosis but accuracy of 94% for TVS for detecting moderate and severe disease.

**Fig. 13:**
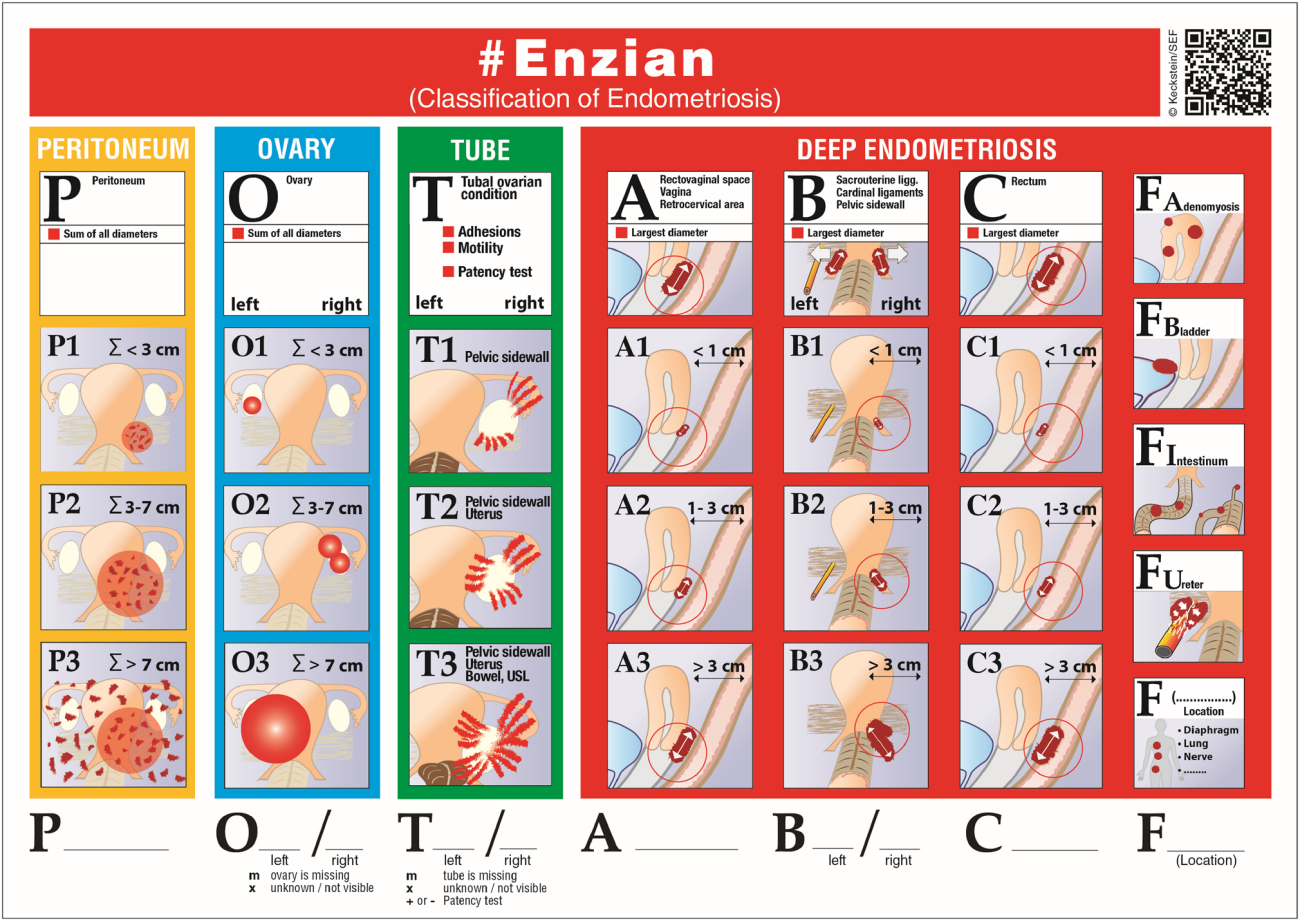
#Enzian classification for the comprehensive description and classification of endometriosis. The individual affected compartments are classified according to the localization and size of the findings using a code. The compartments are marked with capital letters and in the case of paired organs or structures (ovary, tube, USL and ureter); the sides are also shown separately behind the respective letters. The lesions are classified with a code that takes into account both the location and the size of the different findings. The results of soft markers (sliding signs) and tube perturbation (e.g., with HyCoSy) are also shown. The classification can be used for both non-invasive (TVS = (u), MRI = (m)) and invasive ((s) = surgery) diagnostics

To overcome the lack of adequately describing DE, the Enzian classification was developed in 2003 [44, 49] and further extended to ovarian endometriosis and secondary adhesions in 2021 [50] (Fig. 13). Up to date, three studies have evaluated the accuracy of TVS in combination with the Enzian classification. Hudelist et al. evaluated 195 women with DE undergoing TVS and surgery and found good agreement, especially for Enzian compartments A (vagina, rectovaginal space), C (rectum) and FB (urinary bladder. DE in compartments A, B, C, and FB were diagnosed with a sensitivity of 84%, 91%, 92%, and 88%, respectively, and a specificity of 85%, 73%, 95%, and 99% [51]. Enzelsberger [52] classified deep endometriosis preoperatively by one or combined methods (clinical examination, TVS, MRI) using the cEnzian classification. Less accurate results could be explained by a lack of standardized requirements in the classification application and possibly nonvalidated expertise of the different investigators in this study, which is not yet part of the certification requirements for participating centers. The problem of the lack of comprehensive documentation of endometriosis with the available classification systems has been increasingly discussed [53], especially since non-invasive diagnostics have gained considerably in accuracy and are increasingly regarded as a fundamental part of the treatment of patients. Instead of combining different classification systems, a single system such as the #Enzian classification can be used for both non-invasive and invasive diagnostics [50]. Di Giovanni et al. [54] retrospectively investigated 93 patients undergoing TVS and surgery using the #Enzian classification. Sensitivities and specificities for TVS in compartments were between 86 and 100% (Table 1).

**Table 1:** The accuracy of sonographic classification (#Enzian(u)) of endometriosis compared with the result of surgical classification #Enzian(s) (retrospective study by di Giovanni A. et al. [54] and prospective study of Montanari E. [19])

	di Giovanni et al.		Montanari et al.	
	*n* = 93 (retrospective)		*n* = 745 (prospective)	
#Enzian compartment	Sensitivity (%)	Specificity (%)	Sensitivity (%)	Specificity (%)
O left	100 (92–100)	96 (86–100)	90 (86–94)	96 (94–98)
O right	100 (87–100)	98 (87–100)	89 (84–92)	98 (96–99)
T left	^*^(87–93)	^*^(82–90)	90 (87–93)	86 (82–90)
T right	^*^(84–91)	^*^(87–93)	88 (84–91)	90 (87–93)
A	97 (90–100)	86 (64–97)	95 (92–96)	93 (89–96)
B left	97 (90–100)	70 (47–87)	91 (88–93)	88 (83–93)
B right	100 (95–100)	90 (70–99)	83 (79–87)	94 (91–96)
C	100 (92–100)	96 (86–100)	93 (90–95)	95 (92–98)
FB	86 (42–100)	100 (96–100)	94 (87–98)	100 (99–100)
FI	100 (80–100)	100 (95–100)	50 (41–59)	99 (98–100)
FU	100 (75–100)	100 (95–100)	78 (63–89)	100 (99–100)
FO	100 (48–100)	98 (92–100)	57 (37–76)	100 (99–100)

*O* = ovary; *T* = adhesions of the adnexa; *A* = vagina, rectovaginal septum, torus uteri; *B* = USL, parametrium; *C* = rectum; *FB* = bladder; *FI *= intestinal above the rectosigmoid (> 16 cm from the anus); *FU* = ureteral obstruction; *FO* = other lesions

*For #Enzian compartment T, it was not possible to calculate sensitivities and specificities, as there were too few cases without any lesion during TVS or surgery

**Fig. 14:**
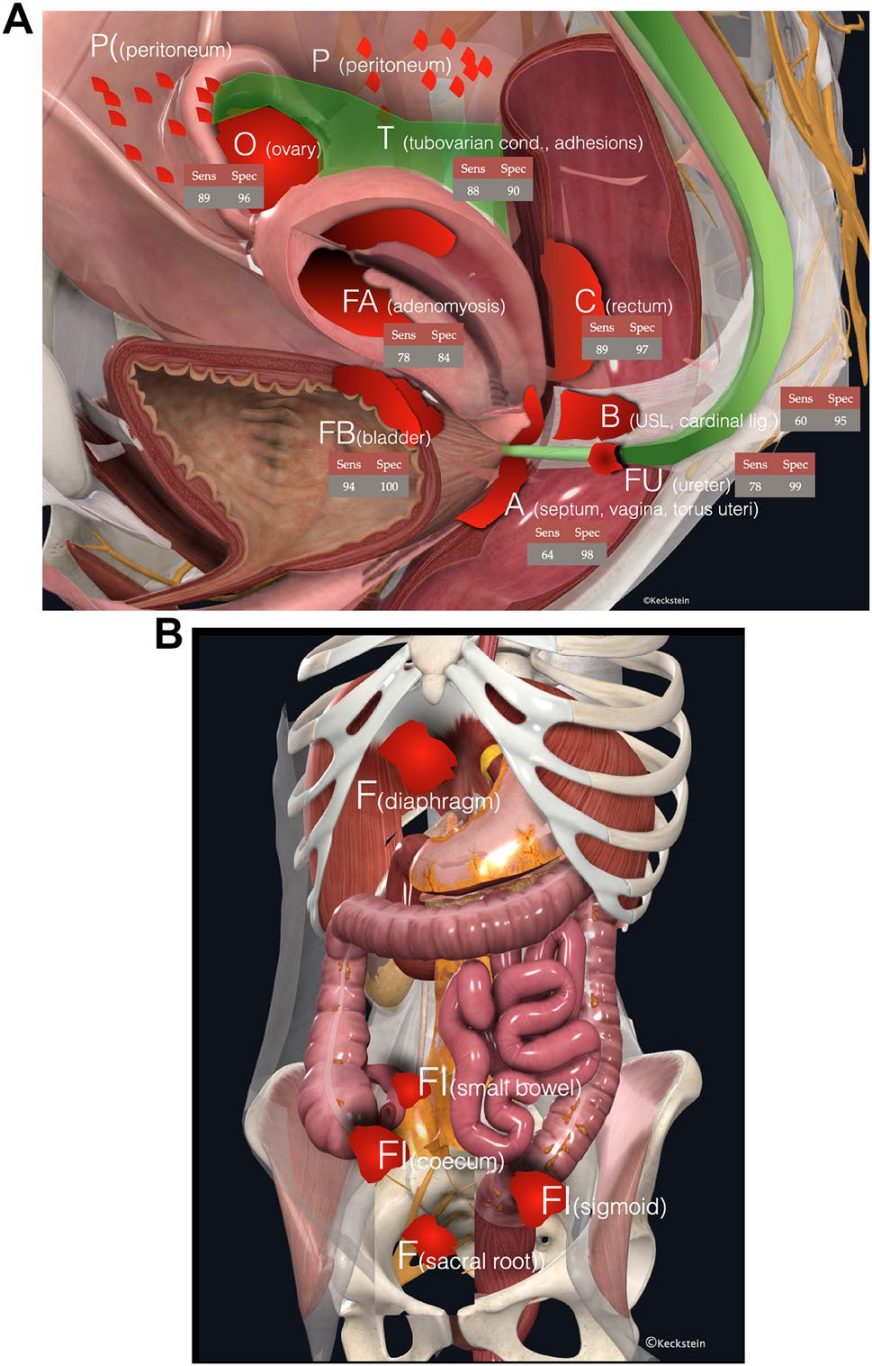
**A** The different compartments (P, O, T, A, B, C, FA, FB, and FU) in the pelvis subdivided according to the #Enzian classification, including data on the accuracy of TVS in the diagnosis of endometriosis. (sens. = sensitivity, spec. = specificity). Data derived from meta-analyses and individual studies show a large variance, which can be explained by different investigators and, in some cases, not yet standardized examination techniques. This is confirmed by the fact that TVS has a very high specificity in most compartments. The data are given in %. References: O [4, 6, 19], T [6, 19], A [5, 19, 36], B [5, 19, 36], C [19, 24, 35, 55], FA [39], and FU [19]. **B** Visualization of the other F compartments (except FA, FB, and FU) according to the #Enzian classification. To date, there are insufficient valid data on the accuracy of sonographic diagnostics in these compartments

Recently, a prospective, multicentre study including 745 patients undergoing TVS in combination with the #Enzian classification and surgery [19] documented sensitivities for the detection of DE ranging from 50% (#Enzian compartment FI—other intestinal locations) to 95% (#Enzian A), specificities from 86% (#Enzian T left) to 99% (#Enzian FI) and 100% (#Enzian FB—urinary bladder, FU—ureters and FO—other extragenital locations) with positive predictive values of 90% (#Enzian T right) to 100% (#Enzian FO), negative predictive values of 74% (#Enzian B left) to 99% (#Enzian FB and FU) and accuracies of 88% (#Enzian B right) to 99% (#Enzian FB). These data support that DE can be accurately evaluated using TVS in combination with the #Enzian classification (Table 1 and Figs. 13, 14a, b)).

Therefore, the ISGE recommends the best possible detection of endometriosis using the systematic IDEA criteria and the comprehensive classification by the #Enzian classification [13].

### Transvaginal sonographic examination by an experienced examiner is not inferior to MRI diagnostics in sensitivity and specificity in the prediction of the extent of deep endometriosis

Several meta-analyses confirmed the equivalence of TVS and MRI in the diagnosis of the specific pelvic anatomic location of endometriosis lesions [17, 55, 56]. Prospective studies to compare TVS and MRI in the diagnosis of endometriosis are rare. Indrielle-Kelly et al. assessed the diagnostic accuracy of TVS and MRI in preoperative pelvic DE mapping on the same cohort of 51 patients, using 1 standardized IDEA-based protocol [23, 57]. They found that TVS and MRI were similar in their performance in endometriosis mapping. The dynamic aspect of ultrasonography combined with the high-resolution transvaginal ultrasound probe increases the detection rate of the obliteration of the pouch of Douglas and the overall accuracy of the ultrasound. Due to the non-superiority of MRI in most anatomic localizations, its better availability, and lower cost, TVS is recommended as the method of the first choice. MRI examination is superior to ultrasound for technical/physical reasons, especially in cases of exclusive pelvic wall involvement, possibly involving nerves, diaphragm, and/or lung. Furthermore, it should be mentioned that the #Enzian score is also applicable to MRI, but minor modifications are suggested [58, 59].

### The significant advantage of non-invasive imaging and classification of endometriosis is the differentiated planning or possible avoidance of radical surgical interventions

The risk factors for surgical complexity and postoperative complications after more or less radical colorectal surgery in DE are well known. The lesion's location and size significantly impact this [60, 61]. For example, in intestinal endometriosis, the height of surgical anastomosis [62], the extent of parametrial involvement and the surgical technique [63] are essential factors. Similarly, ureteral and parametrial involvement or the combination of different pelvic DE lesions influences both symptoms and the expected complexity of surgical treatment. Proper preinvasive recognition of disease extent and #Enzian classification using sonography or MRI can help to ensure an accurate assessment of both the indication and the anticipated surgical procedure [57, 64]. Both improve patient counseling and the planning of interdisciplinary procedures, if necessary. For example, TVS has been shown to correctly determine the size of colorectal DE before surgery [65]. Aas-Eng and colleagues have also demonstrated that TVS correctly reflects the distance between colorectal DE lesion and the anal verge and adequately estimates the height of the final surgical anastomosis [66], which is important for risk assessment. Rectal endoscopic sonography RES [55, 67], although an alternative for determining the location and extent of the lesion, requires appropriate gastroenterological expertise and cannot be used to assess other pelvic structures.

The risk for surgical complications correlates with the extent of lesions and, therefore, with a higher Enzian/#Enzian score in certain anatomic compartments. For example, Poupon et al. developed a nomogram classification [68] showing a direct correlation between complication risk and Enzian classification. Similar observations were made by Nicolaus et al. [69].

Therefore, the use of TVS for non-invasive assessment of surgical complexity and risk factors for surgical complications is recommended.

## Conclusions and perspective

The use of TVS is an efficient and accurate tool for the non-invasive diagnosis of endometriosis. Although this imaging technique does have limitations such as operator dependency, it is cost-effective and enables the clinician to establish a diagnosis in cases of the ovarian and deep disease. Many doctors still rely on surgical and histological confirmation as a gold standard test. This approach is highly questionable and may not be up to date. The authors advocate for using of TVS as a primary tool to evaluate women with suspected endometriosis and to stratify these patients into low- and high-risk patients based on the results of TVS-based preoperative assessment. A nearly complete non-invasive diagnosis of endometriosis opens up new perspectives for conservative and surgical treatment. Using the #Enzian classification, also in the context of sonographic assessment, provides clinicians with a uniform “language” for a comprehensive and easily reproducible description of endometriosis.
